# Synthesis and Characterizations of PdNi Carbon Supported Nanomaterials: Studies of Electrocatalytic Activity for Oxygen Reduction in Alkaline Medium

**DOI:** 10.3390/molecules26113440

**Published:** 2021-06-05

**Authors:** Muhammad Sufaid Khan, Rozina Khattak, Abbas Khan, Qiuling Chen, Jan Nisar, Zahoor Iqbal, Abdur Rashid, Abdul Waheed Kamran, Ivar Zekker, Muhammad Zahoor, Khalid J Alzahrani, Gaber El-Saber Batiha

**Affiliations:** 1 Department of Chemistry, University of Malakand, Chakdara 18800, Pakistan; arhamiqbal2017@gmail.com (Z.I.); waheedkamran1989@gmail.com (A.W.K.); 2 Department of Chemistry, Shaheed Benazir Bhutto Women University, Peshawar 25120, Pakistan; rznkhattak@yahoo.com; 3 Department of Chemistry, Abdul Wali Khan University, Mardan 23200, Pakistan; abbas80@awkum.edu.pk; 4 Material Science and Engineering Department, Henan University of Technology, LianhuaRoad 100, Zhengzhou 450001, China; chenqiuling@haut.edu.cn; 5 National Centre of Excellence in Physical Chemistry, University of Peshawar, Peshawar 25120, Pakistan; jan_nisar@uop.edu.pk; 6 Hydrogeochemistry Laboratory, Department of Environmental Sciences, Faculty of Biological Sciences, Quaid-i-Azam University, Islamabad 45320, Pakistan; rashid.a2010@yahoo.com; 7 Institute of Chemistry, University of Tartu, 50090 Tartu, Estonia; ivar.zekker@ut.ee; 8 Department of Biochemistry, University of Malakand, Chakdara 18800, Pakistan; mohammadzahoorus@yahoo.com; 9 Department of Clinical Laboratories Sciences, College of Applied Medical Sciences, Taif University, P.O. Box 11099, Taif 21944, Saudi Arabia; ak.jamaan@tu.edu.sa; 10 Department of Pharmacology and Therapeutics, Faculty of Veterinary Medicine, Damanhour University, Damanhour 22511, Egypt; gaberbatiha@gmail.com

**Keywords:** alkaline medium, fuel cells, oxygen reduction reaction, stability of NPs

## Abstract

Electrocatalytic materials offer numerous benefits due to their wide range of applications. In this study, a polyol technique was used to synthesize PdNi nanoparticles (NPs) with different percent atomic compositions (Pd = 50 to 90%) to explore their catalytic efficiency. The produced nanoparticles were characterized using X-ray diffraction (XRD) and electrochemical investigations. According to XRD measurements, the synthesized NPs were crystalline in nature, with crystallite sizes of about 2 nm. The electrochemical properties of the synthesized NPs were studied in alkaline solution through a rotating ring-disk electrode (RRDE) technique of cyclic voltammetry. The PdNi nanoparticles supported on carbon (PdNi/C) were used as electrocatalysts and their activity and stability were compared with the homemade Pd/C and Pt/C. In alkaline solution, PdNi/C electrocatalysts showed improved oxygen reduction catalytic activity over benchmark Pd/C and Pt/C electrocatalysts in all composition ratios. Furthermore, stability experiments revealed that PdNi 50:50 is more stable in alkaline solution than pure Pd and other PdNi compositions.

## 1. Introduction

PEMFCs (polymer electrolyte membrane fuel cells) are a promising alternative energy source for transportation and stationary applications. The advantages of fuel cell technology include a high conversion rate, a natural operating temperature, environmental friendliness, and high stability [1]. According to the literature, PEMFCs can have the same potential power as batteries, power grids, and combustion engines [2]. Despite the fact that PEMFCs are relatively cost-effective energy sources, their market availability for widespread use faces some obstacles that should be resolved. The oxygen reduction reaction (ORR) on the cathode of the fuel cell, proceeds with a slower chemical kinetics, which is the most noticeable drawback of this technology [3]. A number of oxygen-containing species such as OH^−^, O_2_^−^, HO_2_^−^ and H_2_O_2_ are produced in aqueous media during the process which compete with oxygen reduction on electrode surface involving adsorption and desorption processes [3]. Due to their immense importance in storage devices and electrochemical energy conversion systems, the oxygen reduction reaction on the cathode is extensively studied and still research in this field is in progress. Apart from its scientific significance, oxygen electrocatalysis is a requirement of industry where the renewable energy devices rely on oxygen electrochemistry (fuel cells, fuel synthesis, and metal-air batteries). As mentioned above the systems based on oxygen electrochemistry, the ORR has been documented as slow reaction kinetically as mostly platinum or platinum based electrode used are contaminated that affect the efficiency of oxygen reduction reaction. Pt is also an expensive metal with low availability around the globe. As a result, scientists have begun developing Pt-free nanoparticles as a dopant material for such electrodes, which has increased the catalytic activity and stability of cathode materials [4,5,6].

It is thought that combining transition metals with Pd improves its catalytic ability for oxygen reduction. Palladium alloys containing other transition metals have shown increased oxygen reduction efficiencies [7,8,9]. The addition of transition metals to Pd alloys alters the structural properties of the developed alloy, and the electronic bands of Pd exhibit different electrical charge transportation efficiency as a result of these structural changes [10,11].

To develop an efficient electrode for oxygen reduction, transition metal such as nickel (Ni) was added to palladium in various ratios in the current study and tested for its oxygen reduction capabilities. The polyol method was used to prepare PdNi electrocatalysts with different atomic ratios, which were then supported on carbon. The nanoelectrocatalysts that were synthesized are thought to be able to reduce oxygen molecules and would have a higher stability than previously reported expensive electrocatalysts. The structural and physical behaviors of the prepared samples were determined using XRD. Because the support material was carbon rather than an oxide or a mix of oxides, FTIR is not a primary technique for the PdNi supported on carbon. The morphology of materials can be determined using TEM or HRTEM. We did not employ TEM or HRTEM because our main focus is on electrocatalysis rather than morphology. The oxygen reduction activity of the prepared materials was calculated by electrochemical measurements using the RRDE process. The alkalinity of medium was maintained with dilute sodium hydroxide (NaOH) during the experiments to compare the oxygen reduction activities of the catalysts in alkaline medium and check their stabilities.

## 2. Results and Discussion

### 2.1. Structural and Electronic Properties of PdNi/C Electrocatalysts

#### X-ray Diffraction of PdNi/C Electrocatalysts

**Figure 1:**
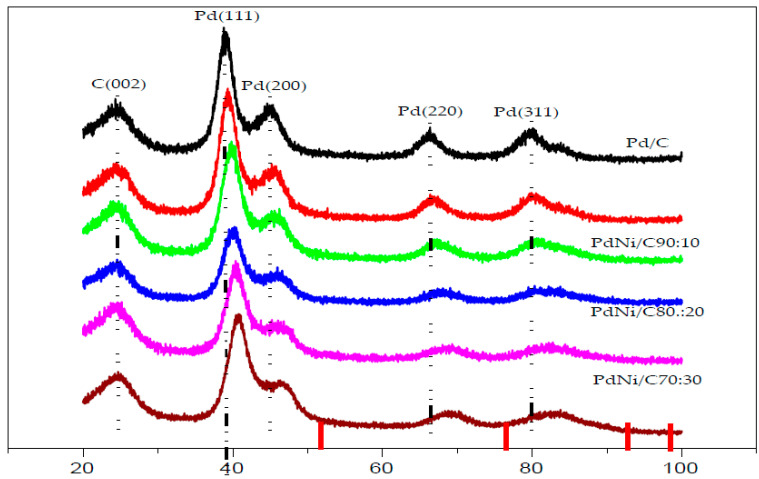
X-ray diffraction data for carbon-supported Pd and PdNi/C catalysts.

X-ray diffraction (XRD) was used to characterize the structural properties of the Pd/C and PdNi/C catalysts, and the results are presented in Figure 1. The usual signs of Pd face centred cubic (fcc) structure can be seen in the XRD pattern. In the XRD pattern of any of the PdNi catalysts, the signals show the characteristic peaks of the fcc structure of Pd. In general, the main peaks of PdNi/C lie between the signals of Pd/C and pure Ni, 2θ in degrees are indicated at the bottom of Figure 1 (red lines) nickel (PDF #04-0850) [12]. This can be considered evidence of alloy formation between Pd and Ni. Figure 1 clearly shows five peaks: The peak at 2θ around 25 degrees; the diffraction peak corresponds to the indices (002) indicating the planes of carbon Vulcan XC-72. The other four peaks for Pd/C appeared at around 39° (Pd (111)), 46° (Pd (200)), 67° (Pd (220)) and 80° (Pd (311)) also confirm the presence of Pd in the prepared alloy. Similar result has been reported in the literature [13,14,15].

**Figure 2:**
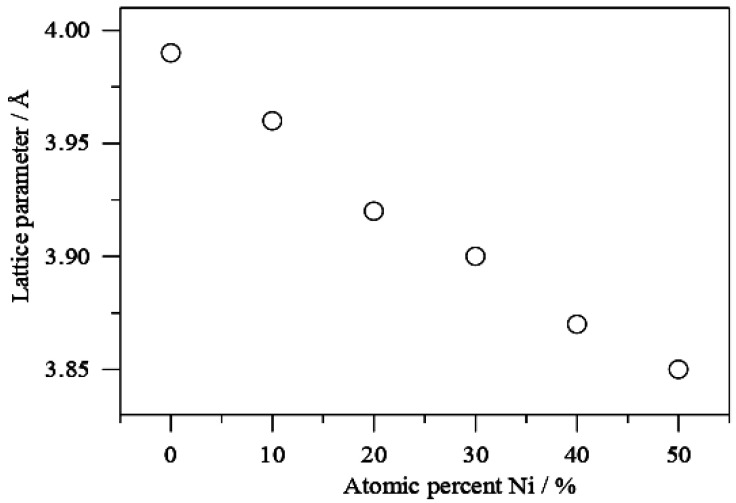
Variation of the lattice parameters vs percentage of Ni in PdNi/C alloys.

When comparing the signals for PdNi/C to the reference monometallic Pd/C catalyst, the signals for all proportions are sequence wise shifted toward higher values of 2θ. This change in peak position is commonly interpreted as evidence of alloy production, which results in lattice contraction. When compared to the other materials including reference Pd, the shift in 2θ towards higher values is more pronounced in the PdNi/C 50:50 material, most likely due to a higher incorporation of the second metal, which leads to a smaller lattice spacing. The inclusion of Ni also resulted in a reduction in particle size, as demonstrated by the widening of the peaks. These findings are consistent with those seen in the literature [16]. The lattice parameter indicating structural properties were calculated with Equations (1)–(3) and their values are given in Table 1. Pd/C, PdNi/C (90:10), PdNi/C (80:20), PdNi/C (70:30), PdNi/C (60:40), and PdNi/C (50:50) had mean crystallite sizes of 2.9, 2.5, 2.1, 1.9, 1.8, and 1.7 nm, respectively, computed from the Pd (220) diffraction peaks (Table 1). These approximated results revealed the effect of the second metal Ni in PdNi/C alloys. Furthermore, PdNi/C nanoparticles have lower lattice parameter values than Pd/C nanoparticles, showing that the metals (PdNi) have been alloyed [12]. When the Ni percentage in PdNi/C catalysts is increased, the lattice parameters are found to decrease [16]. The fluctuation of lattice properties with Ni content is shown in Figure 2.

Scherrer’s Equation (1) was used to compute the average crystallite size of the materials.

(1) $$D=\frac{0.9\lambda }{\omega \cos\theta }.\;$$

In the preceding Equation (1), *λ* is the incident radiation’s wavelength, *ω* is the peak width at half intensity, and *θ* is the highest intensity position. Because the particles are assumed to be spherical, the factor 0.9 emerges. The peak with indices 220 of the Pd fcc structure was chosen in the computations because the broad carbon peak has no effect in this region. The apparent lattice parameter at (220) diffraction and the corresponding Pd-Pd distance were also estimated.

Equation (2) was used to obtain the lattice constant.

(2) $$a_{exp}=\frac{\sqrt{2}\theta }{sin\theta }\;.$$

The inter-atomic distance (dPd-Pd) between two palladium atoms was estimated in the following way.

(3) $$d_{fcc}=\frac{\sqrt{2}}{2}a_{exp}.\;$$

Table 1 shows that the lattice constant falls as the atomic percentage of Ni increases in all PdNi/C ratios, demonstrating the creation of an alloy between Pd and Ni [16]. Furthermore, when comparing PdNi/C alloys to reference Pd/C, the reduction in lattice parameter revealed a lattice contraction due to the partial replacement of Pd sites in PdNi/C alloys by smaller Ni atoms. A similar finding has already been reported in the literature [17]. The decrease in Pd-Pd distance for all of the prepared PdNi/C catalysts is explained by changes in the lattice parameter.

**Figure 3:**
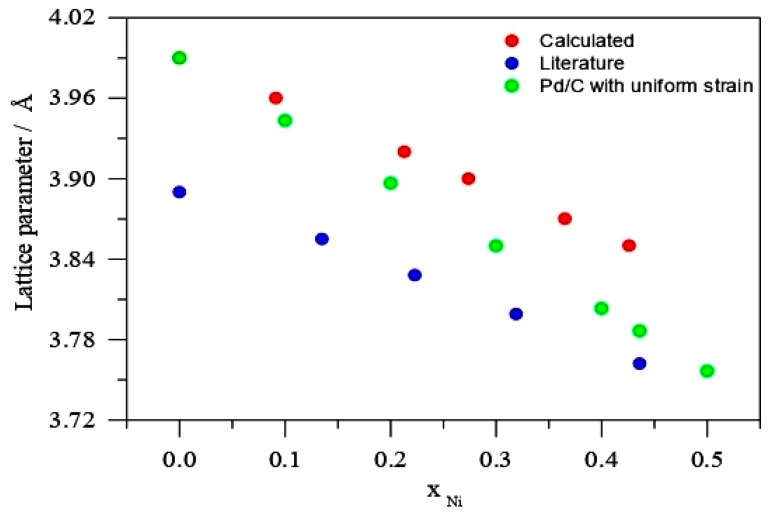
Lattice parameter against Ni molar fraction calculated from Equation (5), Reference [19] and calculated Pd uniform strain *a*_0_ (3.970 Å).

Figure 3, displays the Vegard’s law plot as given by Equations (4) and (5):

(4) $$\chi_{M}=\frac{a-a_{0}}{a_{M}-a_{0}}\;.$$

In Equation (4), *a* represents the proportion-dependent experimental value of the lattice parameter for PdNi/C electrocatalysts, *a*_M_ is the lattice parameter for a PdNi solid phase, and *a*_0_ is the lattice parameter of pure metal, Pd. Table 2 shows the computed lattice parameters for the PdNi/C theoretical composition. According to Equation (4), alloy and pure elements must have the same crystal network as of Pt, however this is rarely the case. As a result, using the Pt_M_ solid phase as a reference alloy will be more accurate. Consequently, in Equation (4), the atomic ratio of the new element (α) should be supplied as a correction factor, and the updated equation can now be stated as Equation (5), which is used to estimate the atomic fraction of the second metal Ni in this situation [18].

(5) $$\chi_{M}=\alpha \frac{a-a_{0}}{a_{M\;}-a_{0}}$$

Figure 3 and Table 2 illustrate a comparison of the literature value [19] and our projected values.

According to Figure 3, the lattice parameter of PdNi solutions is dependent on the Ni content, as evidenced by literature [19], with PdNi/C solid phase lattice parameter (*a*_alloy_ 3.762) at Ni molar proportion of 0.436 and Pd/C lattice parameter (*a*_0_ 3.990). Furthermore, the resulting XRD data were plotted with PdNi solid solution data described in the literature. Its figure revealed that a lattice constant of 3.86 corresponds to a PdNi alloy with a Ni content of roughly 11% [19]. It can be concluded from Figure 3 and Table 2 that the uniformed strain fugitive Ni fraction from the alloy phase in PdNi/C (90:10, 80:20, 70:30, 60:40, and 50:50) is around 11%. The molar fraction (Ni) will be larger if Ni is included in the Pd/C lattice parameter. Figure 3 also shows a curve of uniform Pd strain with lattice parameter (*a*_0_ = 3.970) for further comparison. The uniform strain reduces as the percent of Ni increases in the calculation from reference Pd to PdNi solid solution (*a*_alloy_ = 0.436) devoid of any strain. For various Pd-based alloys, a comparable conclusion has been examined [20].

**Figure 4:**
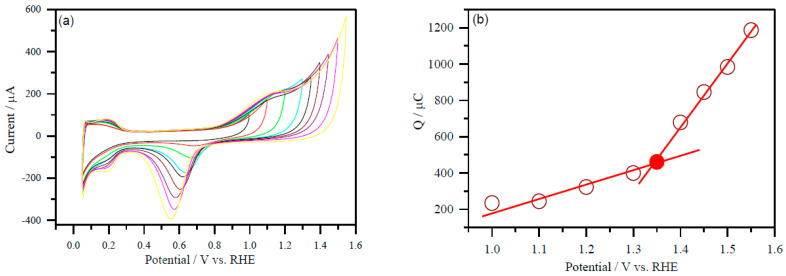
A representative plot to determine the active surface area of the Pd and PdNi (50:50) carbon supported electrocatalysts. (**a**) CVs with different upper potential limits at a scan rate of 50 mV s^−1^; PdNi/C 50:50 (**b**) the integrated charge as a function of the upper potential limit. The value of the charge of the inflexion point (colored) was used to determine the ESA. CVs were performed in Ar-purged 0.5 M H_2_SO_4_ solution.

A representative Figure 4a displays a selected set of CVs of Pd/C and PdNi/C electrocatalysts recorded in 0.5 M H_2_SO_4_ with varied upper potential limits ranging from 1V to 1.650 V (vs. RHE) for the computation of electrochemical active area of PdNi/C (50:50). This probable potential range has been chosen based on the literature [21]. The oxide reduction peak has been seen to increase with the stepwise increase of potential ranges from 1 to 1.650 V (vs. RHE). Furthermore, cyclic voltammograms revealed that an increase in potential corresponds to a higher oxide reduction peak for each material.

Furthermore, the integrated charge (after double layer correction) for the reduction of oxygen-covered Pd surface was plotted as a function of the upper potential limit from 1 to 1.650 V as given in Figure 4b (PdNi/C 50:50). The coordinates at the point where the straight line changes its slope (potential/V versus Q/C) correspond to the formation of a complete PdO monolayer when the reference is taken into account. In Figure 4b (PdNi/C 50:50), colour dot represents the value of the charge associated with the inflexion point.

The different values of the inflexion points were recognized as the charge corresponding to the formation of a complete PdO monolayer for different proportion of Pd/Ni. The reference charge Q/μC was computed from the value of Pd per unit surface area, which is in close agreement with the literature value, that is, 420 μC/cm^2^ for the Pd [22].

As a result of the PdO monolayer completion approach, the electrochemical active area of Pd/C and PdNi/C has been determined. In comparison to Pd/C, the inflexion point in PdNi/C 60:40 and PdNi/C 50:50 is shifted to lower charge values in the continually rising potentials. This could be due to the second metal, Ni, interfering. For DC, a similar finding has been observed [22].

The electrochemical active area was determined using the following Equation (6) based on the reduction oxide charges of each material vs. potential, assuming that 420 μC charges had completely covered the surface of the Pd/C based on the literature.

(6) $$\mathrm{PdO}\;\mathrm{Area}=\left(Q/\mu C/420{\mu \mathrm{Ccm}}^{-2}\right).$$

Table 3 lists the estimated values of electrochemical active surface area (ESA) of Pd/C and PdNi/C with various percent atomic compositions. The active area reduces as the percent of Pd in Pd/Ni decreases, according to the findings. For example, the computed area of pure Pd/C is 3.45 cm^2^, while it is roughly 1.17 cm^2^ for the PdNi/C 50:50 ratio. Furthermore, the Pt/C electrochemical active area was determined using the hydrogen adsorption/desorption region in cyclic voltammetry as well as Equation (6) and shown in Table 3 [23].

### 2.2. Cyclic Voltammetry of Pt/C, Pd/C and PdNi/C in Alkaline Solution

**Figure 5:**
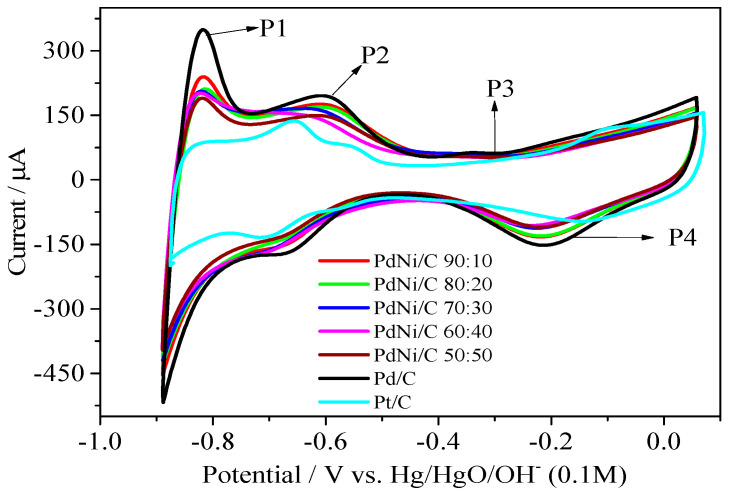
Cyclic voltammetry curves (5th cycle) of the Pt/C, Pd/C and PdNi/C catalysts in argon-saturated electrolyte, 0.1 M NaOH. Sweep rate: 0.05 V s^−1^, where: P represents peak.

Electrochemical properties of the electrocatalysts; Pt/C, Pd/C and PdNi/C with its different compositions were studied by cyclic voltammetry in 0.1 M NaOH solution saturated with argon. Initially, four cycles were conducted to clean the surface of the materials using a potential range of −0.876–0.474 V (vs. Hg/HgO/OH^−^), and then normal CVs were measured at a scan rate of 0.05 V s^−1^ in a potential range of −0.876 to 0.074 V (vs. Hg/HgO/OH^−^). Figure 5 shows a comparison of typical current-potential curves.

In alkaline solution, different processes were observed in CVs. The Pd/C curve on the positive scan reveals three current peaks linked to different electrochemical processes occurring on the catalyst surface. Peaks P1 and P2, with potential ranging from −0.876 to −0.576 V (vs. Hg/HgO/OH^−^), may be attributed to the oxidation of hydrogen adsorbed (and absorbed) as shown in Equation (7).

Pd-H_abs/ads_ + OH^−^→ Pd + H_2_O + e.^−^ (7)

The generation of the Pd(II) oxide layer can be attributed to peak P3, which appears above −0.326 V (vs. Hg/HgO/OH^−^). The electrochemical mechanism of this oxidation process is unknown. It is believed that OH^−^ ions are chemisorbed first in the early stages of oxide formation, followed by the formation of higher valence oxides at higher potentials, as shown below (Equations (8)−(10)).

Pd + OH^−^ ↔ Pd-OH_ads_ + e^−^ (8)

Pd-OH_ads_ + OH^−^ ↔ Pd-O + H_2_O + e^−^ (9)

Pd-OH_ads_ + Pd-OH_ads_ ↔ Pd-O + H_2_O. (10)

The hydrogen desorption peak P2 partly overlaps with the OH^−^ adsorption (Equation (8)). The reduction process at about −0.206 V (peak P4) corresponds to the reduction of Pd(II) oxide as mentioned in Equation (11).

Pd-O + H_2_O + 2e^−^ ↔ Pd + 2OH^−^ (11)

Furthermore, as shown in Figure 5, the cyclic voltammograms (CVs) of PdNi/C electrocatalysts were identical to that of Pd/C in Ar-saturated alkaline media, that is, 0.1 M NaOH solution. Pt/C, on the other hand, has a distinct behavior when compared to other materials. Literature [24] has published similar CVs of Pt and Pd supported on graphene sheets. However, two peaks can be seen in the hydrogen adsorption/desorption region (P1 and P2). This suggests that the peaks P1 and P2 for Ni-containing catalysts may be changed positively or negatively, and that this change may be linked to the interference of nickel oxide formation [25]. As the Ni content increases, there is a small shift to lower positive values of the onset potential of the oxides formation in the potential range of about −0.426 to 0.074 V (vs. Hg/HgO/OH^−^), as documented in literature. In comparison to Pd/C and Pt/C, the charge involved in the hydrogen adsorption/desorption region (approximately −0.876 V–0.500 V vs. Hg/HgO/OH^−^) varies among the different composition of PdNi/C electrocatalysts.

Among the PdNi/C varied proportions, PdNi/C 90:10 had the highest hydrogen adsorption/desorption, while PdNi/C 50:50 had the lowest. The oxides phase is initiated at −0.4 V to −0.1 V (vs. Hg/HgO/OH^−^) and the reduction region is between −0.2 V and 0 V (vs. Hg/HgO/OH^−^). The presence of a large amount of Pd in PdNi/C 90:10 compared to the other proportions of the PdNi/C causes this behavior of CVs for the PdNi/C at all proportions compared to the Pd/C. It could be owing to lower Pd atomic ratios in PdNi/C alloys, such as PdNi/C 50:50, and the surface of the catalyst engaged in hydrogen adsorption/desorption and alloy oxidation/reduction (formation/reduction of Pd hydroxide and oxyhydroxide). The alloy formation effect, that is, smaller lattice size in PdNi/C 50:50 compared to Pd/C, could explain the lower hydrogen adsorption/desorption charge. CVs have been shown to behave similarly in alkaline medium [26].

Figure 5 shows that the CV of the hydrogen adsorption/desorption region of Pt/C electrocatalyst is lower than that of Pd/C and PdNi/C electrocatalysts. This is most likely owing to the high concentration of OH^−^ ions in the alkaline solution. These OH^−^ ions are deposited on the surface of the Pt/C by adsorption, inhibiting the hydrogen adsorption active sites. As a result, among all the prepared materials, Pt/C had the lowest hydrogen adsorption/desorption region [27].

### 2.3. Pd/C, PdNi/C and Pt/C Oxygen Reduction Reaction in Alkaline Solution

**Figure 6:**
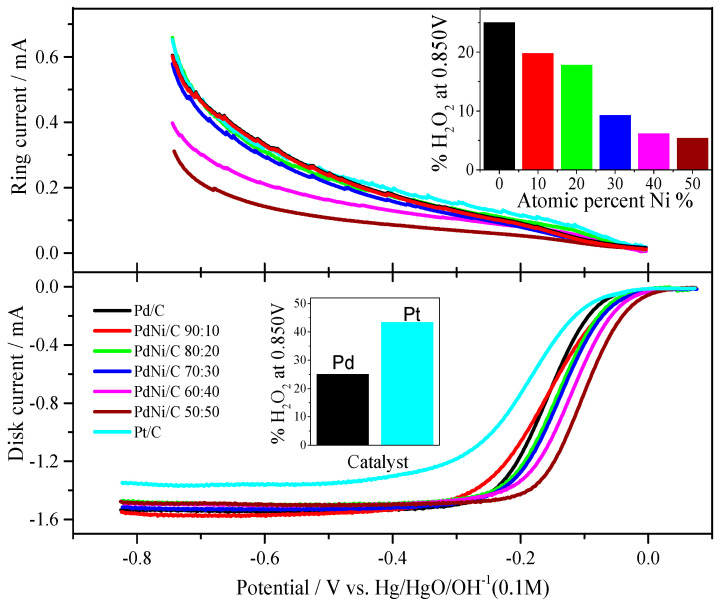
ORR polarization curves of Pt/C, Pd/C and PdNi/C in 0.1 M NaOH solution saturated with oxygen, and ring current corresponding to H_2_O_2_. Rotation rate: 2500 rpm. Sweep rate: 0.005 V s^−1^. (Inset: Production of %H_2_O_2_ by Pd/C and PdNi/C, and comparison of Pd/C with Pt/C).

Figure 6 summarizes the ORR polarization scheme. The ORR experiments were performed in a 0.1 M NaOH medium with a continuous flow of O_2_ gas. Pd/C and Pt/C have had their polarization curves recorded in various rotations [28]. However, differing PdNi/C atomic proportions resulted in distinct limiting currents. On the rotating ring disc electrode, Pd/C and PdNi/C show moderate variances in limiting current, however Pt/C has a substantial divergence in limiting current. Furthermore, as the fraction of Ni grows, the potential range of kinetic-diffusion controlled current densities expands. On the ring electrode, hydrogen peroxide is also detected (Figure 6). Hydrogen peroxide is identified in the potential range of about −0.1 V (vs. Hg/HgO/OH^−^) and continues to occur up to −0.826 V. The polarization curves of the Pd/C, PdNi/C, and Pt electrodes were compared at 2500 rpm.

Several traits emerge during the experimentation process. When compared to Pd/C and Pt/C, the potential region of mixed kinetic diffusion control on PdNi/C is deviated towards higher potentials, indicating that the second metal, Ni, is helping in the most active surface for the ORR in alkaline solution. In terms of hydrogen peroxide production, a lesser quantity is always formed on the PdNi/C (all proportions), whereas the generation of H_2_O_2_ detected a bigger amount on the reference Pd/C and homemade Pt/C. In the literature, similar results have been reported for thin Pd films supported on Pt and Pd-based alloys [29,30,31,32]. In addition, all ORR curves had a well-defined diffusion-limiting current region between −0.8 and −0.2 V (vs. Hg/HgO/OH^−^) and a mixed kinetic-diffusion control region from −0.2 to 0.05 V (vs. Hg/HgO/OH^−^). When compared to the benchmarks Pd/C and Pt/C, the mixed kinetic-diffusion control region is higher for all PdNi/C ratios, especially for the PdNi/C 50:50. In comparison to Pd/C and Pt/C, PdNi/C electrocatalysts (all compositional ratios) enhance ORR activities in alkaline solution.

The kinetic current has been calculated using the ORR polarization curves by considering the mass-transport correction using the Koutecky-Levich Equation (12) to explore the electro activity of the PdNi/C, Pd/C, and Pt/C in detail. The kinetic currents were computed in alkaline solution at 0.074 V (vs. Hg/HgO/OH^−^) for all materials.

(12) $$\frac{1}{i}=\frac{1}{\mathrm{ik}}+\frac{1}{i_{L}}$$

Measured current, kinetic current, and diffusion-limited current are represented by i, ik, and i_L_, respectively. To compare the mass and specific area activities of the catalysts, the diffusion-limited current was standardized against and then normalized to the mass and active area of Pd or Pt. PdNi/C 90:10, PdNi/C 80:20, PdNi/C 70:30, PdNi/C 60:40, and PdNi/C 50:50 have computed specific activity of 0.07, 0.11, 0.16, 0.29, and 0.62 mA cm^−2^, respectively. Specific activity for reference Pd/C and Pt/C electrocatalysts were determined at 0.02 and 0.03 mA cm^−2^, respectively. ORR was used to calculate the specific activities of all materials, which are listed in Table 3. On the basis of these estimated values of specific activities, it is determined that all PdNi/C alloys, particularly PdNi/C 50:50, have higher catalytic activities than home-made Pd/C and Pt/C. The presence of Ni accounts for the greater specific activity of the PdNi/C 50:50. When compared to Pd/C and Pt/C, it improves catalytic activity, consequently. Changes in electronic structure caused by the addition of a second metal alter the catalytic activity of PdNi/C. In the literature, hollow Pd^1^Ni^1^/C has been shown to improve catalytic activity in an alkaline environment [33,34].

The amount of hydrogen peroxide generated during the ORR could potentially be measured using the rotating ring-disk electrode (RRDE) approach. The ORR can be determined by two electrons or four electrons based on the amount of H_2_O_2_ relative to the amount of O_2_ reduced (Figure 6, inset: Comparison of the production of %H_2_O_2_). Equation (13) [35] can be used to compute the proportion of H_2_O_2_ in percent.

(13) %H2O2=100·(2·irNid+irN)

where i_r_ is the current measured in the ring, i_d_ is the current measured on the disk and N is the collection efficiency of the ring-disk electrode, which depends on the dimensions of the gap between the disk and the ring [36]. Figure 6 and Table 3 show that the production of H_2_O_2_ is higher in the case of Pd/C and Pt/C but reduces as the amount of Ni in the catalyst increases, implying that the addition of Ni favors the ORR via near 4 electrons. Figure 6 depicts the production of hydrogen peroxide in the given potential profile. In comparison to Pd/C and Pt/C, Figure 6 and Table 3 show that ORR catalytic activity of all proportions of PdNi/C, specifically PdNi/C 70:30, PdNi/C 60:40, and PdNi/C 50:50, is around 90.74%, 93.82%, and 94.60% conversion of O_2_ molecules to OH^−^, respectively, reflecting almost 4 electrons reaction mechanism. It can be seen that Ni enhances ORR in alkaline solutions as well, and that the 4-electron path is strongly favored. The H_2_O_2_ percentages are summarized in Figure 6 and Table 3. For all atomic ratios of PdNi/C, the percent of H_2_O_2_ decreases, especially for PdNi/C 50:50 (5.40%) compared to Pd/C (25.03%) and Pt/C (43.35%). In comparison to PdNi/C, the significant production of H_2_O_2_ in Pd/C and Pt/C might be attributed to maximum adsorption on the surface of Pd/C and Pt/C, where all active sites are finished for readsorption [37].

To summarize, ORR polarization curves for Pd/C, Pt/C, and PdNi/C electrocatalysts indicate that the electrochemical reaction is led by kinetic and mixed activation-diffusion control across almost the entire potential range. Increased oxygen transport causes an increase in current as rotation rates increase. Considering the low levels of H_2_O_2_ found (Figure 6), it appears that the ORR is primarily a four-electron transfer process leading to water production (Equation (14)) [29,38,39].

O_2_ + 4H^+^ + 4e^−^ → 2H_2_O. (14)

The reason for the number of transported electrons is presented below in terms of Levich graphs. The Pt/C ORR, on the other hand, is going through two electrons and producing a high amount of H_2_O_2_.

### 2.4. Determination of the Kinetic Parameters of Pd/C, PdNi/C and Pt/C from ORR in Alkaline Solution

In general, the exchange current density of a catalyst for an electrochemical reaction determines its activity. Since determining this parameter for technical electrodes is difficult and uncertain, another method of measuring catalyst’s activity in fuel cells is the mass activity (A_m_) according to Equation (15) [40].

(15) $$A_{m}=\frac{i_{0.9}}{W},$$

where i_0.9_ is the current density in mA/cm^2^ at 0.9 V, and W is the loading metal (usually Pt) in mg/cm^2^. The value of 0.9 V was chosen to prevent any concentration polarization from being used. The mass activity is provided for operation with reactants of 100%O_2_ and 100%H_2_ [40]. With a geometric area of 0.247 cm^2^ and a load of 30 μg of metal per cm^2^, the mass of 7.43 μg of Pd and 5.71 μg of Pt on the Pd/C and Pt/C working electrodes were determined. Since the reaction on Pd catalyst is slower than on Pt catalyst, the current (i) value at 0.85 V was used in this study.

**Figure 7:**
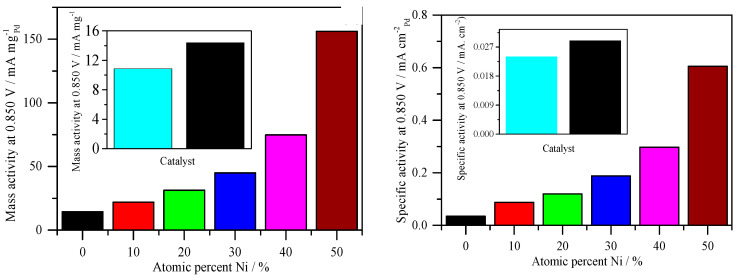
Catalytic activities derived from Figure 6. (**Left**): Mass activity with different percent atomic ratio of Ni (Inset: Comparison of the mass activity of Pd and Pt). (**Right**): Specific activity with different percent atomic ratio of Ni (Inset: Comparison of the specific activity of Pd and Pt). Rotation rate: 2500 rpm. Sweep rate: 0.005 V s^−1^.

It is typical to express mass activity as kinetic current at any given potential normalized by the mass of the catalytic metal for electrodes other than functional cathodes. In the case of PdNi atomic ratios, mass activity was determined by dividing the ORR kinetic current at 0.85 V by the mass of Pd, while in the case of Pt mass activity, Pt mass was used (Figure 7) [40]. The mass activities of pure Pd/C and Pt/C catalysts are lower than all PdNi/C catalysts, according to this study, and the mass activity increases as the percentage of Ni increases. Table 3 shows that the observed patterns are in strong alignment with numerical values. Clearly, the molar ratio of the two metals influences the mass activities of the PdNi/C electrocatalysts. This sort of normalization indicates the economic relevance of the prepared PdNi/C catalysts because the catalyst’s performance is also quantified in terms of kW/$ [40]. Aside from mass activity, specific activity, defined as the kinetic current normalized by the electrochemical active surface area (ECSA) [40], is another effective technique to compare activity. The current per unit area, which is a genuine physicochemical parameter, is obtained using this method of normalization. PdNi/C 50:50 has the highest specific activity, which is more than six times that of Pd/C and more than seven times that of home-made Pt/C, according to ECSA-normalized currents (Figure 7). Overall, the findings show that adding Ni to Pd not only increases ORR mass but also leads to higher specific activity, which is consistent with previous findings for PdNi nanoparticles in the literature [32,41]. This increase in catalytic activity could be due to changes in particles’ surface structure or a more favorable particle size, as has been demonstrated for Pt nanoparticles [29].

**Figure 8:**
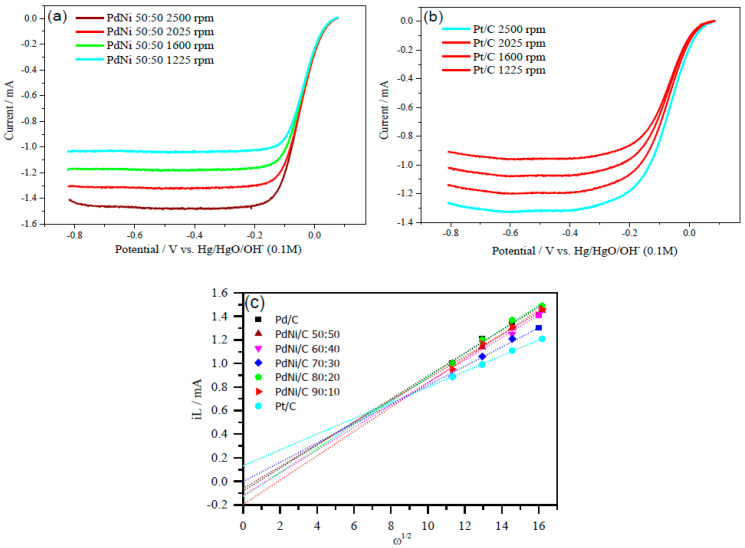
ORR polarization curves of (**a**) PdNi/C 50:50 and (**b**) Pt/C, at various rotation rates (2500, 2025, 1600 and 1225 rpm). (**c**) Levich plots derived from the ORR curves of the PdNi/C at different compositions.

Aside from mass and specific activities, the Levich plot, which explains the electron transfer mechanism of the reaction, is another essential parameter. The Levich plots computed the number of electrons required for ORR in an alkaline medium in two or four electrons. Data from varied rotation rates are shown in Figure 8 (2500, 2025, 1600 and 1225 rpm). The limiting current (*i_L_*) vs. square root of *ω* provides straight line with an intercept, which is related to the limiting current at zero rotation rate of the electrode in the Levich-plot. The ORR polarization curves at −0.076 V potential (vs. Hg/HgO/OH^−^) were used to draw the Levich-plots (Figure 8c). The Levich Equation (16) [37] was used to calculate the number of electrons involved per O_2_ molecule reduction.

(16) $$i_{L}=0.62nFD^{3/2}v^{-1/6}\omega^{1/2}C_{O2}^{0}$$

$$∴B=0.62nFD^{3/2}v^{-1/6}C_{O2}^{0}.$$

In Equation (16), *i_L_* expresses diffusion-limiting current, *ω* is the angular velocity and related to the rotation rate of the electrode, *n* is the transferred electron number, *F* is Faraday constant (F = C mol^−1^), diffusion coefficient is *D* of O_2_ (O_2_ = 1.9 × 10^−5^ cm^2^ s^−1^), kinetic viscosity is 0.01 cm^2^ s^−1^, and C° is the bulk concentration of O_2_ (1.2 × 10^−6^ mol cm^−3^). The constant 0.2 is used to convert rotation rate to rotations per minute. The slope(s) of the straight line(s) (Levich-plot(s)) in Figure 8c reveals B factor for PdNi/C between 0.093 and 0.103 mA/rpm, Pd/C 0.098 mA/rpm, and Pt/C 0.067 mA/rpm. The number of electrons was computed and reported in Table 4 based on the value of B. For Pd/C and PdNi/C, the estimated transferred number of electrons ranged from 3.49 to 3.86, whereas Pt/C was 2.49 at around −0.2 V (vs. Hg/HgO/OH^−^). The computed electron values show that a four-electron (4e^−^) pathway precedes the ORR from −0.076 to 0.074 V (vs. Hg/HgO/OH^−^) and that O_2_ is reduced to OH^−^ during the reaction. By using a two-electron transfer mechanism, the Pt/C dominates in the creation of more hydrogen peroxide (H_2_O_2_). As shown in Figure 6, the four electron transfer mechanism elaborates the low formation of H_2_O_2_ on PdNi/C, which is then readsorbed on the active sites of Pd in PdNi/C. When H_2_O_2_ is entirely adsorbed on the catalysts’-skin active sites, the second metal offers active sites for additional H_2_O_2_ adsorption [37].

Finally, the estimates revealed that the ORR process uses roughly four electrons, except in the case of Pt/C. This behavior of PdNi/C electrocatalysts reveals that hydrogen peroxide production is reduced at about 4 electrons mechanism. The large creation of hydrogen peroxides at Pt/C is due to the fact that the ORR in alkaline occurs via two-electron pathway [39]. In the literature, two-electron pathway of Pt/C (Pt, 20% weight ratio) for ORR have been reported [42].

**Figure 9:**
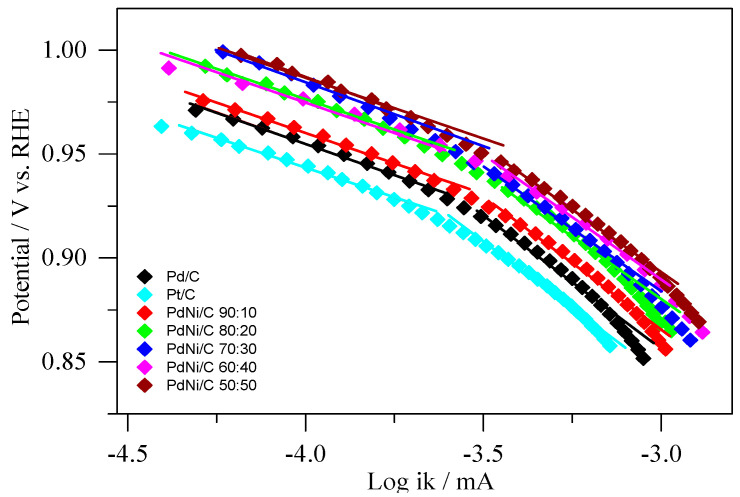
Tafel plots of kinetically controlled ORR currents on the Pd/C, Pt/C, and PdNi/C; 0 V vs. RHE = −0.926 V (vs. Hg/HgO/OH^−^) at 2500 rpm was used to convert to the RHE (reversible hydrogen electrode) scale.

For ORR in 0.1M NaOH, Tafel plots of the mass transport corrected currents (ik) were generated (Figure 9). Pd/C, Pt/C, and PdNi/C electrocatalysts have two distinct areas with corresponding two values of the Tafel slopes. Equation (12) was used to determine the kinetic currents (ik). When comparing the kinetic current densities at −0.076 V (vs. Hg/HgO/OH^−^), the PdNi/C (all compositions) appear to be significantly more active for the ORR than the Pd/C and Pt/C, indicating an activity improvement. In addition, as shown in Figure 9, there is a linear relationship between log ik and the applied potential (E/V) for all materials. Table 4 shows the Tafel slopes, which were recorded in the applied potential region of mixed kinetic-diffusion control for prepared materials. The slopes were found between (57 mV dec^−1^, 62 mV dec^−1^) and (119 mV dec^−1^, 120 mV dec^−1^) in low and high overpotential ranges, respectively. The adsorbed ion (OH^−^) may be responsible for a small deviation in Tafel slopes for Pd/C, Pt/C, and PdNi/C 90:10 at low overpotential [43]. In principle, the tangent at each point has a different slope. However, at a rotation rate of 2500 rpm, the observation yields values in the possible region of 0.850 V < E < 0.985 V (vs. Hg/HgO/OH^−^) for a rough estimate of the range of the Tafel slopes.

### 2.5. Accelerated Stability Tests in Alkaline Solution

**Figure 10:**
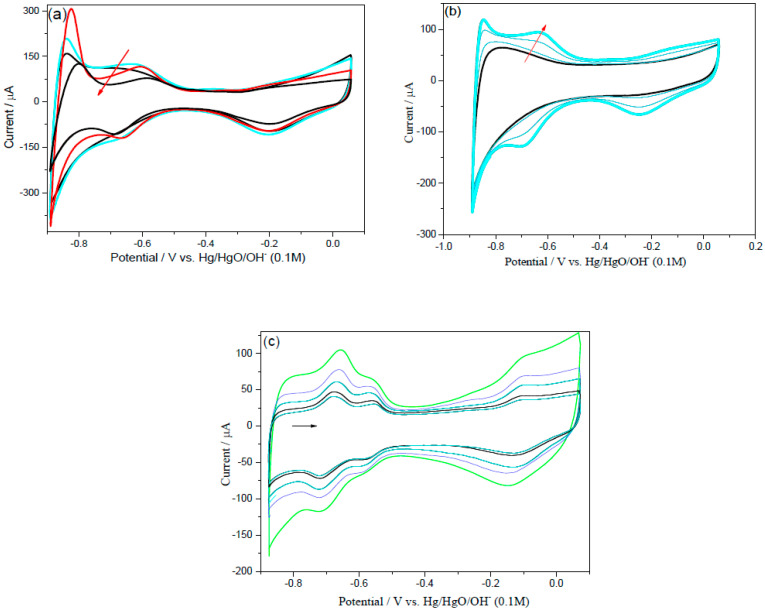
Stability test CVs, for Pd/C (**a**), PdNi/C 50:50 (**b**) and Pt/C (**c**) catalysts at 1000 potential cycles in 0.1 M NaOH from −0.876 to 0.074 V vs. Hg/HgO/OH^−^.

To examine the electrochemical stability of the prepared materials, the nanoparticles were electrochemically cycled in 0.1 M NaOH in the presence of Ar gas for a long period of time, that is, 1000 continuous cycling at 0.050 V/s with the potential profile between −0.876 V and + 0.074 V (vs. Hg/HgO/OH^−^). The voltammograms (CVs) for Pd/C, PdNi/C 50:50, and homemade Pt/C are shown in Figure 10a–c. After long-term stability, PdNi/C 50:50 performed well as active electrocatalyst, as shown in Figure 10b. Interestingly, the active region of Pd/C and PdNi/C 90:10 increases in the first 250 to 300 cycles and then gradually decreases. It’s possible that this is due to the roughness and cleanliness of the catalyst’s surface [39]. This activity of Pd/C and PdNi/C 90:10 suggests that alkaline solutions help to rearrange the surface of Pd atoms during the cycling process, resulting in more active sites. Similar findings for PdCu porous materials in acidic solution have been published in the literature [22]. Furthermore, the Pd/C and PdNi/C 90:10 ratios continued to degrade, indicating less stability. This finding may be explained by the formation and dissolution of oxides/hydroxides of the respective materials [39]. Surprisingly, after 250 potential cycles, the hydrogen adsorption desorption regions of PdNi/C 80:20, PdNi/C 70:30, PdNi/C 60:40, and PdNi/C 50:50 are continuously increased, implying an increase in the electrochemical active area. ORR polarization curves were also tested in the first five cycles and after 1000 cycles of the stability test. According to the ORR results, activity increases after 1000 cycles as the percent of Ni increases, while it decreases for Pd/C and Pt/C. The continuous enhancement of active regions of the CVs may be due to the roughness of the catalyst’s surface, according to these findings. The shift of hydrogen adsorption/desorption regions was used to assess the growth of the catalyst-skin roughness [44]. Chun-Hua Cui et al. [44] published the results for PtNi/C with a description of the dissolution in acidic medium of the second metal, Ni, and how the Pt-Ni surface could adjust for better catalytic performance and build coordinated active surface sites on catalysts. These findings corroborate our findings for PdNi/C in alkaline solution. Similarly, during this long-term stability test, the active area of all materials is continuously increased after the initial 5 to 1000 cycles and raises the active area. On the other hand, there is no such influence on the Pt/C. This impact may be due to the second metal’s surface cleaning and stability, namely Ni. The CV shapes after electrochemical cycling were consistent with what would be anticipated for a Pd-based material in alkaline solution [38].

In addition, the electrochemical active area generated by the charge passing through the hydrogen absorption area is complicated by hydrogen absorption into the Pd. Figure 10a,c show that Pd/C, PdNi/C 90:10, and Pt/C had low catalytic efficiency and substantial loss of active area after potential cycling, retaining only around 25%, 40%, and 10% of their initial ECSA, respectively. PdNi/C 80:20, PdNi/C 70:30, PdNi/C 60:40, and PdNi/C 50:50, on the other hand, retained and increased the stability of their initial ECSA by 100%, 120%, 150%, and 200%, respectively. This demonstrates that the electrocatalysts with higher Ni percent have better electrochemical stability than Pd/C, PdNi/C 90:10, and Pt/C electrocatalysts, which all have low electrocatalytic activity in alkaline medium.

**Figure 11:**
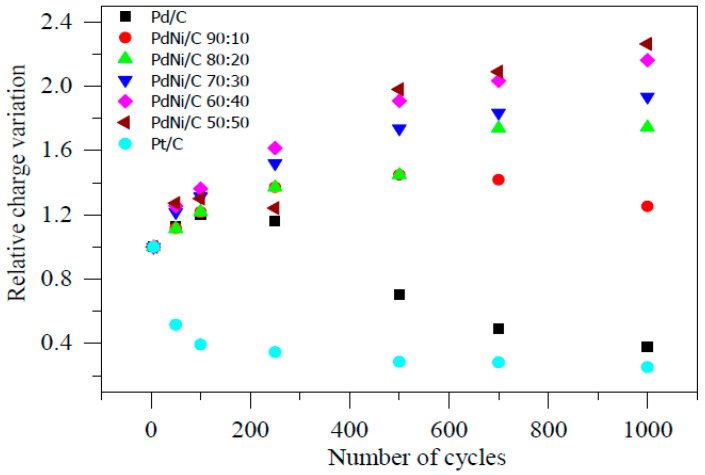
Relative charge variations during accelerated stability tests in 0.1 M NaOH.

Figure 11 also shows the relative charge of Pd/C, PdNi/C (different proportions), and Pt/C. Figure 11 followed the previous discussion in terms of overall Ni stability (PdNi/C 50:50 and PdNi/C 60:40). Although other electrocatalysts had similar electrocatalytic activities prior to the stability test over 1000 potential cycles, this one is obviously more resistant to ECSA loss under potential cycling and thus a more promising ORR electrocatalyst in alkaline solution. In the literature, stability tests for PdCu nanodendrites in acidic medium have been discovered [21].

## 3. Experimental Section

### 3.1. Preparation of Materials

In the dioctylether, nanoparticles of PdNi supported on carbon (PdNi/C) at varied atomic ratios were produced. Metallic precursors of Pd(II) acetylacetonate (Sigma Aldrich: St. Louis, MO, USA) and Ni(II) acetylacetonate (Aldrich) were employed in these experiments. Metal precursors, reduction reagent 1, 2-hexadecanediol, and dioctylether were mixed in a conical flask. The resulting mixture was heated to 110 °C with a constant supply of an inert gas. During the experiment, two compounds were added to control agglomeration, that is, oleylamine and oleic acid. To create nanoparticles (NPs), the temperature of this process was increased until it reached reflux (298 °C). Black colored PdNi NPs were formed, which were subsequently cooled in an open atmosphere. Following that, these NPs were supported on carbon (Vulcan XC-72, Cabot) in the presence of n-hexane and stirred for 12 h. PdNi NPs were washed with different solutions after filtration, including double distilled water, acetone, and ethanol. The washed NPs were dried in an oven for two hours at 80 degrees Celsius [11]. All of these nanoparticles had a metal content of 20% by weight. A similar method was used for preparing a pure Pd/C to be used as a reference material.

### 3.2. Characterizations

#### X-ray Diffraction (XRD)

The X-ray experiments were conducted in a Rigaku equipment, model D Max 2500PC, in the range of 20 to 100 degrees in 2 theta, scan rate of 1 degree min^−1^, and incident radiation wavelength of 1.5406 Å (Cu K α). The lattice constant and average crystallite size were investigated using XRD findings.

### 3.3. Electrochemical Measurements

#### Electrode Preparation

An ultra-thin layer of the catalyst was deposited on a glassy carbon electrode (0.247 cm^2^ geometric area). The resulting electrode was employed as working electrode in the electrochemical measurements. For this, the appropriate amount of catalyst powder was suspended in 1 mL of isopropanol containing 15 μL of Nafion solution for 15 min under continuous ultrasonic stirring. The complete suspension of the catalyst powder resulted in a black ink, which was then applied on the glassy carbon electrode. With the help of a micro-syringe, a suitable volume of suspension was applied to create a layer with a load of 30 μg cm^−2^ of active metal. After the formation of an ultra-thin layer of catalyst over the electrode surface, it was hydrated with ultra-pure water.

### 3.4. Cyclic Voltammetry (CV) and Oxygen Reduction Reaction (ORR)

A Pine CBP potentiostat and a three-electrode electrochemical cell were used to make the electrochemical measurements. The working electrodes were a rotating ring-disk electrode (for ORR and CV) and a glassy carbon disk with a layer of the catalyst deposited on its surface (for accelerated stability test). The auxiliary electrode was a platinum wire, whereas the reference electrodes were mercury/mercury oxide (vs. Hg/HgO/OH^−^) and reversible hydrogen electrode (RHE).

Cyclic voltammetry was used to investigate the general electrochemical behavior by recording cyclic voltammograms (CVs). CVs were recorded in alkaline solutions for all prepared electrocatalysts. In alkaline medium, the CV studies were conducted in the potential range of −0.876 to 0.074 V (vs. Hg/HgO/OH^−^ electrode), which is comparable to (0.050–1 V vs. RHE) at a scan rate of 50 mV s^–1^. The experiments were carried out in argon-saturated solutions of 0.1 M NaOH. The rotating ring-disk electrode (RRDE) technique was used to measure polarization curves in O_2_ saturated alkaline solution to assess catalytic activity for the ORR. A ring-disk electrode with a platinum ring (0.186 cm^2^ geometric area) around a glassy carbon disk (0.247 cm^2^ geometric area) was employed as the working electrode, and a catalyst layer was deposited on the disk electrode.

The polarization curves were collected in alkaline medium at a sweep rate of 0.005 mV s^–1^ in the potential range of −0.876 to 0.074 V (versus. Hg/HgO/OH^−^). The amount of H_2_O_2_ generated was determined by the oxidation current on the ring, while the amount of oxygen reduced was determined by the current generated by the reaction on the disk. The RRDE was operated at different rotation speeds (1225, 1600, 2025, 2500 rpm). These experiments were carried out in oxygen-saturated 0.1 M NaOH solutions.

### 3.5. Determination of the Electrochemical Active Real Surface Area

The real surface area of the Pt can be determined using standard cyclic voltammetry because only hydrogen adsorption happened on the surface of the Pt electrocatalyst. However, due to adsorption and absorption on the surface of the Pd electrocatalysts, the same method is inconvenient for calculating the true surface area of the Pd electrocatalysts. As a result, calculating electrochemical surface active area (ECSA) from hydrogen adsorption/desorption peaks in the potential deposition region is difficult. Because of the uncertain chemical surface nature of the PdNi/C alloys, the CO stripping approach is likewise ineffective. Consequently, the reduction charges of Pd oxides were used to determine the electrochemical active area of all synthesized nanomaterials.

CV measurements were first carried out in the potential range of 0.050 to 1 V vs RHE in order to evaluate the real surface area of Pd electrocatalysts. The experiments were performed by adjusting the higher positive potential limit with varied potential intervals (0.050 V and 0.025 V) up to 1.650 V to identify the potential where one monolayer of Pd-oxide is created. These tests were carried out at a scan rate of 50 mV s^−1^ in a 0.5 M H_2_SO_4_ solution that was kept saturated with argon (Ar). For each potential limit, a new catalyst layer was applied. The charges were estimated by integrating the PdO reduction peaks for each experiment performed up to a specific voltage limit. The palladium oxide monolayer completion was used to measure the electrochemical active area of the prepared nanomaterials.

### 3.6. Stability Test

For a cathode fuel cell, one of the most important characteristics of the electrocatalyst is stability. In order to study long-term accelerated stability testing in both acid and alkaline media, 150 cycles in acidic medium and 1000 cycles in alkaline medium were swept. The continuous potentials between 0.05–1 V (vs. RHE) and −0.876 to 0.074 V (vs. Hg/HgO/OH^−^) were measured at a scan rate of 0.050 V s^–1^ in Ar saturated 0.5 M H_2_SO_4_ and 0.1 M NaOH media. The effect of degradation on electrochemical active area and other kinetic parameters was measured before and after the stability test in both acidic and alkaline solutions, whereas ORR catalytic activity was measured using a linear sweep voltammetry in an electrolyte continuously bubbled with oxygen. A Pine CBP potentiostat was used in all electrochemical studies.

## 4. Conclusions

The oxygen reduction reaction was carried out in alkaline solution in the presence of nanoelectrocatalysts; Pd/C, PdNi/C (in five different atomic ratios of both metals) and Pt/C. The physical and electrochemical properties of the synthesized series of catalysts were thoroughly investigated. XRD data confirmed the alloy formation between the Pd and Ni metals with a crystalline nature and approximately 2 nm crystallite sizes. In all ratios, the catalytic efficiency of PdNi/C was better than the benchmark Pd/C and Pt/C. Among the different ratios of the PdNi/C, the catalytic activity of PdNi/C 50:50 was significantly higher than that of homemade Pd/C and Pt/C and its other ratios. Because the ORR proceeded by a four-electron mechanism in alkaline medium, the formation of H_2_O_2_ was low when PdNi/C catalysts were utilized. The presence of Ni in the alloy, which increased the ECSA for hydrogen peroxide adsorption, was confirmed by CV curves. With a decrease in H_2_O_2_ output, all PdNi/C catalysts outperformed Pd/C and Pt/C in terms of specific and mass activities for ORR. PdNi/C catalysts have shown stability over Pd/C and Pt/C, with up to 1000 cycles in alkaline solutions. To summarize, PdNi/C electrocatalysts outperform standard electrocatalysts in terms of catalytic activity and stability in an alkaline medium, proving to be a cost-effective and stable elecrocatalyst for ORR in an alkaline medium.

## Figures and Tables

**Table 1:** Structural characteristics of carbon-supported Pd/C and PdNi/C nanocatalysts derived from XRD data.

Catalyst	Crystallite Size (nm)	Lattice Constant (Å)	Pd-Pd Distance (Å)
Pd/C	2.91	3.988	2.820
PdNi/C 90:10	2.53	3.956	2.797
PdNi/C 80:20	2.12	3.921	2.773
PdNi//C 70:30	1.90	3.898	2.756
PdNi/C 60:40	1.84	3.871	2.737
PdNi/C 50:50	1.65	3.845	2.719

**Table 2:** Molar composition of Pd/C and PdNi/C nanocatalysts derived from XRD data.

Catalyst	Molar Composition Calculated	Nominal Composition
Pd/C	100	100
PdNi/C 90:10	91:09	90:10
PdNi/C 80:20	79:21	80:20
PdNi//C 70:30	73:27	70:30
PdNi/C 60:40	63:37	60:40
PdNi/C 50:50	57:43	50:50

**Table 3:** ORR activities in Alkaline solutions for carbon-supported Pd, PdNi and Pt catalysts.

Catalyst	Mass Activity (mA/mg) at 0.85 V	%H_2_O_2_ at 0.85 V	Specific Activity (mA/cm^2^) at 0.85 V
Pd/C	14.40	25.03	0.02
PdNi/C 90:10	22.00	19.82	0.07
PdNi/C 80:20	31.39	17.82	0.11
PdNi/C 70:30	45.05	9.26	0.16
PdNi/C 60:40	74.76	6.18	0.29
PdNi/C 50:50	156.63	5.40	0.62
Pt/C	10.86	43.35	0.03

**Table 4:** Data derived from the Levich slopes (B), number of electrons per O_2_ molecule, and Tafel slopes of the ORR for the different catalysts in 0.1 M NaOH.

Catalyst	B Slope (mA)	Number of Electrons	Tafel Slope Lower Region (mA/dec)	Tafel Slope at Higher Region (mA/dec)
Pd/C	0.098	3.65	57	120
PdNi/C 90:10	0.093	3.49	59	120
PdNi/C 80:20	0.096	3.58	60	120
PdNi/C 70:30	0.101	3.76	62	120
PdNi/C 60:40	0.101	3.79	59	120
PdNi/C 50:50	0.103	3.86	60	120
Pt/C	0.067	2.49	58	119
